# The Riot Act

**Published:** 1902-12

**Authors:** 


THE RIOT ACT.—It is past comprehension and belief that any self respecting physician will ignore and fail to pay a small subscription bill of one, two, three dollars, long past due, for his favorite medical journal. Yet it is a fact that there are in Texas many well known and respectable medical men who "pass up" my little bills, month after month, and some of them, year after year, notwithstanding the bills are rendered every few months with a request—rubber stamped—"Be good, do it now." Dr. Lanphear, the editor and publisher of the American Journal of Surgery and Gynecology, one of the best journals published, writes me that he has hundreds of subscribers in Texas, who will not pay him. It is a black eye for the Texas profession and I blush for them. I am now sending out every bill on my books, and I want them paid. I give "value received" and it takes money to pay the printer, to say nothing of buying shoes and stockings for Betty and the baby. I am drawing drafts on the most delinquent, from two years arrears, up, and I hope they will be paid. Most of my old and many—very many new subscribers pay promptly, and I thank them cordially. It is not the small sum due by any one subscriber, but the aggregate of hundreds of subscription bills that induces me to make these drafts and urge the payment of the bills. I earnestly hope that no one will take offense. I know it is a short-crop-year and money is scarce. I am not "hard up," but I need the money in my business. Ante up, boys, and let's have a new *deal*.

DANIEL of the RED BACK.

---

THE ESTABLISHMENT at Washington of a post-graduate Medical School with a special feature of practical instruction in bacteriology under the direction of ex-Surgeon General Sternberg, U. S. A., the ablest and most distinguished bacteriologist in America, is a distinct forward movement. Sanitation, State Medicine, Public Hygiene, as the science is variously called, will be taught, especially. There is at present a big wave of sanitary reform and progress breaking over all countries. The causes of indeginous diseases must be removed. Consumption, typhoid fever, diphtheria, dysentery must be inhibited and done away with. "Sanitation is the medicine of the future," said Prof. Chaille twenty years ago. Read a full prospectus of the new school under the head of "News and Miscellany."

